# Bobbin Tool Friction Stir Welding of Aluminum Thick Lap Joints: Effect of Process Parameters on Temperature Distribution and Joints’ Properties

**DOI:** 10.3390/ma14164585

**Published:** 2021-08-15

**Authors:** Mohamed M. Z. Ahmed, Mohamed I. A. Habba, Mohamed M. El-Sayed Seleman, Khalil Hajlaoui, Sabbah Ataya, Fahamsyah H. Latief, Ahmed E. EL-Nikhaily

**Affiliations:** 1 Mechanical Engineering Department, College of Engineering at Al Kharj, Prince Sattam Bin Abdulaziz University, Al Kharj 16273, Saudi Arabia; moh.ahmed@psau.edu.sa; 2 Department of Metallurgical and Materials Engineering, Faculty of Petroleum and Mining Engineering, Suez University, Suez 43512, Egypt; mohamed.elnagar@suezuniv.edu.eg; 3 Mechanical Department, Faculty of Technology and Education, Suez University, Suez 43518, Egypt; Mohamed.Atia@suezuniv.edu.eg (M.I.A.H.); ahmed.eassa@ind.suezuni.edu.eg (A.E.E.-N.); 4 Department of Mechanical Engineering, College of Engineering, Imam Mohammad Ibn Saud Islamic University, Riyadh 11432, Saudi Arabia; kmhajlaoui@imamu.edu.sa (K.H.); fhlatief@imamu.edu.sa (F.H.L.)

**Keywords:** bobbin tool, AA1050-H14, pin geometry, travel speed, welding temperature, mechanical properties

## Abstract

Bobbin tool friction stir welding (BT-FSW) is characterized by a fully penetrated pin and double-sided shoulder that promote symmetrical solid-state joints. However, control of the processing parameters to obtain defect-free thick lap joints is still difficult and needs more effort. In this study, the BT-FSW process was used to produce 10 mm AA1050-H14 similar lap joints. A newly designed bobbin tool (BT) with three different pin geometries (cylindrical, square, and triangular) and concave shoulders profile was designed, manufactured, and applied to produce the Al alloy lap joints. The experiments were carried out at a constant tool rotation speed of 600 rpm and a wide range of various welding travel speeds of 200, 400, 600, 800, and 1000 mm/min. The generated temperature during the BT-FSW process was recorded and analyzed at the joints’ center line, and at both advancing and retreating sides. Visual inspection, macrostructures, hardness, and tensile properties were investigated. The fracture surfaces after tensile testing were also examined. The results showed that the pin geometry and travel speed are considered the most important controlling parameters in BT-FSW thick lap joints. The square (Sq) pin geometry gives the highest BT-FSW stir zone temperature compared to the other two pins, cylindrical (Cy) and triangular (Tr), whereas the Tr pin gives the lowest stir zone temperature at all applied travel speeds from 200 to 1000 mm/min. Furthermore, the temperature along the lap joints decreased with increasing the welding speed, and the maximum temperature of 380 °C was obtained at the lowest travel speed of 200 mm/min with applying Sq pin geometry. The temperature at the advancing side (AS) was higher than that at the retreating side (RS) by around 20 °C. Defect-free welds were produced using a bobbin tool with Cy and Sq pin geometries at all the travel welding speeds investigated. BT-FSW at a travel speed of 200 mm/min leads to the highest tensile shear properties, in the case of using the Sq pin. The hardness profiles showed a significant effect for both the tool pin geometry and the welding speed, whereas the width of the softened region is reduced dramatically with increasing the welding speed and using the triangular pin.

## 1. Introduction

In the last few years, in engineering applications, the focus is directed more on Bobbin Tool Friction Stir Welding (BT-FSW) due to various advantages over Conventional Tool Friction Stir Welding (CT-FSW) [1]. The name ‘‘bobbin tool; BT’’ refers to the shape of a tool that consists of two shoulders (upper and lower) connected with a pin [2]. In fact, the bottom shoulder in BT design replaced the backing plate used in the CT-FSW [1]. The BT-FSW process starts with a slow speed until plastic deformation starts, followed by increasing travel speed to the required value. The two shoulders generate frictional and deformation heat from both sides to the workpiece during welding [3]. In friction stir welding (FSW) processes, using BT has many advantages over the use of a conventional tool (CT) such as the welded structure is symmetric in thickness, low distortion of weld joint can be obtained, the elimination of root for welds, a backing plate is not required, and high force is not required for fixing the weld plates and possibility welding a hollow section (U and H shapes) [3]. Various designs of the shoulder were suggested and applied in CT-FSW and BT-FSW processes [4]. Flat and concave are the primary design features among all shapes of shoulders [5]. Concentric circles, ridges, grooves, and knurling are secondary features that may be added for welding thick parts [6,7]. For the concave shoulder, the concavity angle is another important parameter to the weld joint properties. Compared with the flat shoulder, the concave shoulder has been demonstrated to be able to minimize weld flash and improve the joint properties [4]. Whilst the tool shoulder stimulates bulk material flow, the stirring pin during the welding process fosters a layer-by-layer material to flow [8]. Furthermore, the heat generation during the FSW has a strong effect on the weld quality, and it comes from specific tool surfaces, designed tool shoulder, and pin geometry [9,10].The geometry of the tool pin affects the flow of plasticized material and the joint efficiency [10,11]. In fact, the pin geometry is an important parameter in the friction stir welding (FSW) process for temperature history, material flow, and grain size, as well as the quality of the FSW joints [12,13]. Fujii et al. [14] investigated the effect of CT-FSW shape (column without threads, column with threads, and triangular prism shape) on mechanical properties of welding 5 mm AA1050-H14-H24 Al alloy in similar butt joints at different rotation and traverse welding speeds and found that column pin without threads offers butt weld joint with best mechanical properties and this tool shape prompted defects less than the other two tools. Chandrashekar et al. [15] carried out CT-FSW on 6 mm Al plates using three pin profiles: tapered, cylindrical, straight square, and straight cylindrical. The results showed that the square pin profile produced better properties for the joints in comparison with the other two pin profiles. Zhao et al. [16] examined the influence of four conventional tools (CTs) having different pin geometries on the AA2024 Al alloy joints quality and reported that the screw-pitched taper pin produced friction stir welds with minimum defects. Elangovan et al. [17] studied the role of five pin profiles: tapered cylindrical, straight cylindrical, threaded cylindrical, triangular, and square to friction stir weld of AA 6061 Al alloy and found that the CT with the square pin geometry produced defect-free welds for all the applied downward forces used. Mahmoud et al. [18] studied the dispersion of SiC in AA1050-H14-H24 Al surface to fabricate surface composites based on friction stir process using four CTs with different pins: triangular, circular with thread, circular without thread, and square. The square pin geometry produced more homogeneous dispersion of the SiC particles than the other pins, whereas the circular pin profile showed more wear resistance than the flat-faced pin profiles. Wen et al. [9] studied the mechanical properties of the BT-FSW 4 mm thick AA2219-T78 Al alloy welds at various welding speeds from 130 to 350 mm/min and reported a maximum tensile strength obtained at 210 mm/min travel speed with joint efficiency 70%. Li et al. [19] investigated the effect of BT-FSW rotation speed on 4 mm thick 6082-T6 butt joints using a smooth cylindrical pin and concluded that the temperature at the retreating side (RS) was higher than the advanced side (AS) by 20 °C. Moreover, the maximum ultimate tensile strength of 262.7 MPa was obtained at a rotation speed of 800 rpm. Wang et al. [20] studied the influence of rotation speed on the microstructure and mechanical properties of 3.2 mm thick AA2198-T851 BT-FSW butt welds using a cylindrical featureless pin and reported symmetrical hardness distribution along the cross-sections for all welds, and the maximum tensile strength of 370 MPa was reached at 800 rpm. In spite of these achievements in CT-FSW parameters, the BT-FSW parameters needs more research to clarify the effect of pin geometry and travel welding speed, especially for a thick weld for a material possesses low deformation resistance to avoid the role of precipices or solution hardening. Thus, the aims of this work are placed on to determine the influence of the pin geometry and the welding travel speed on the heat generation and the joint quality of 10 mm thick AA1050-H14 similar lab joints welded via BT-FSW. The mechanical properties in terms of hardness and tensile properties are also examined and analyzed. 

## 2. Materials and Methods

AA1050-H14 Al-sheets of 5 mm thickness, 1000 mm length, and 1000 mm width were cut into plate specimens having the dimension of 120 mm length and 110 mm width to BT-FSW purposes to produce similar lap joints. Table 1 and Table 2 show the nominal chemical composition and mechanical properties of the as-received AA1050-H14 Al alloy, respectively.

**Figure 1:**
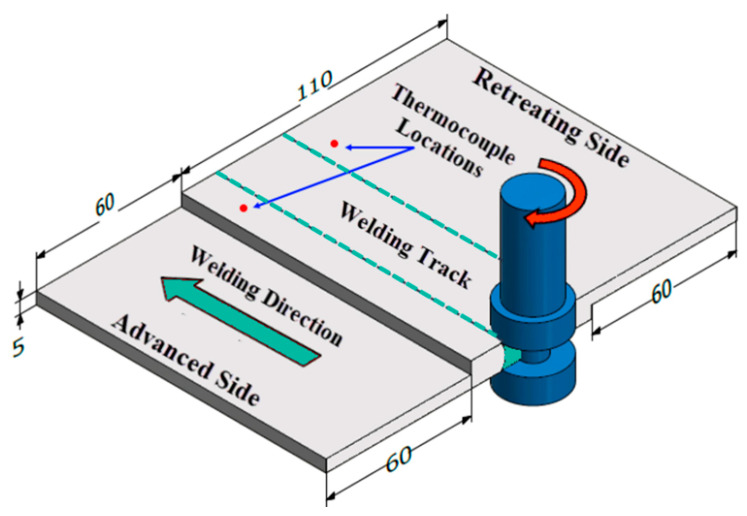
Dimensions of BT-FSW lap joint (all dimensions in mm).

Figure 1 shows example of lap joint arrangement; two sheets 5 mm thick, 100 mm wide, and 120 mm long were used with 40 mm overlap to develop lab joints.

**Figure 2:**
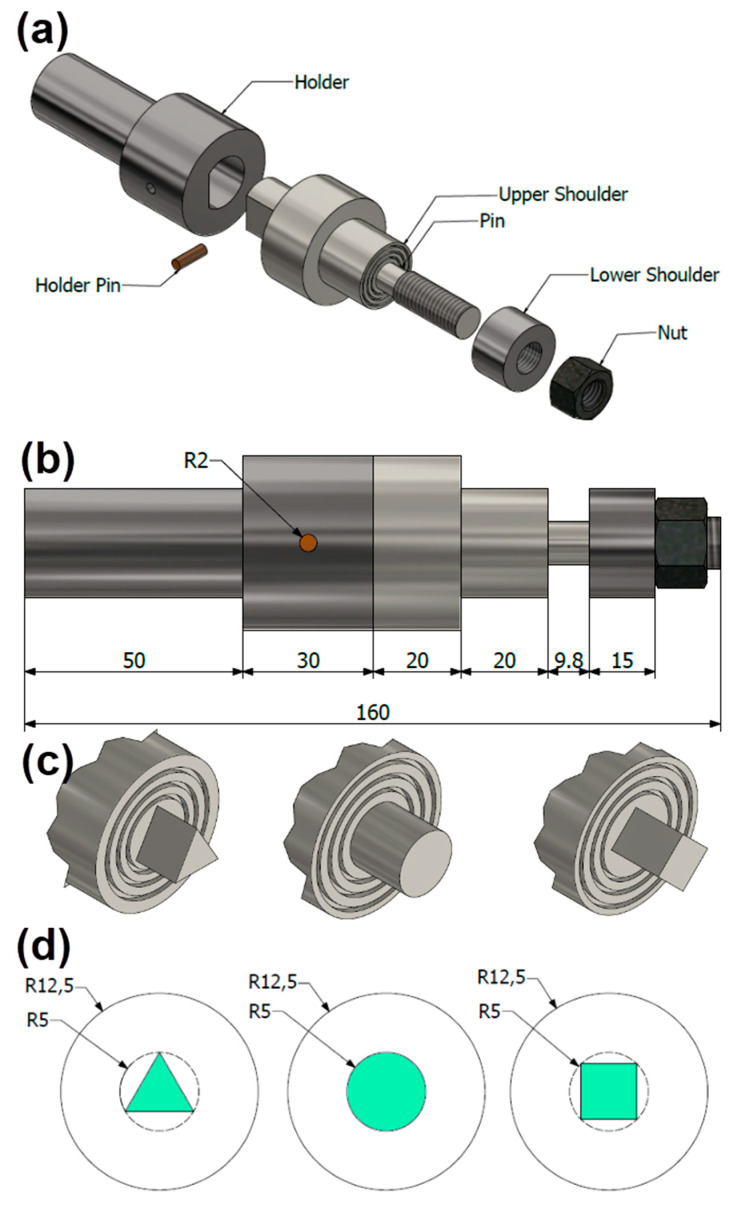
Dimension of BT assembly (all dimensions in mm). (**a**) Exploded assembly of BT parts, (**b**) Dimensions of BT, (**c**) 3D view of used BT pin profiles, and (**d**) dimension of BT pins and shoulders.

A newly designed and fabricated bobbin tool with different pin geometries was tried in the current study to explore the influence of BT-FSW pin geometries on the joint properties. Figure 2a,b shows the dimensions of the bobbin tool assembly and its various parts, upper shoulder, holder, and lower shoulder. The three different pins, cylindrical (Cy), square (Sq), and triangular (Tr) (Figure 2c,d), were used, with the same upper and lower shoulder dimensions. The new bobbin tools with their pin facilities were machined from cold worked H13 tool steel and were heat-treated to obtain hardness of 52 HRC. The shoulders gap is kept constant to be 25 mm. Both shoulders have concave (6°) and cavities features as given in Figure 2c.

**Figure 3:**
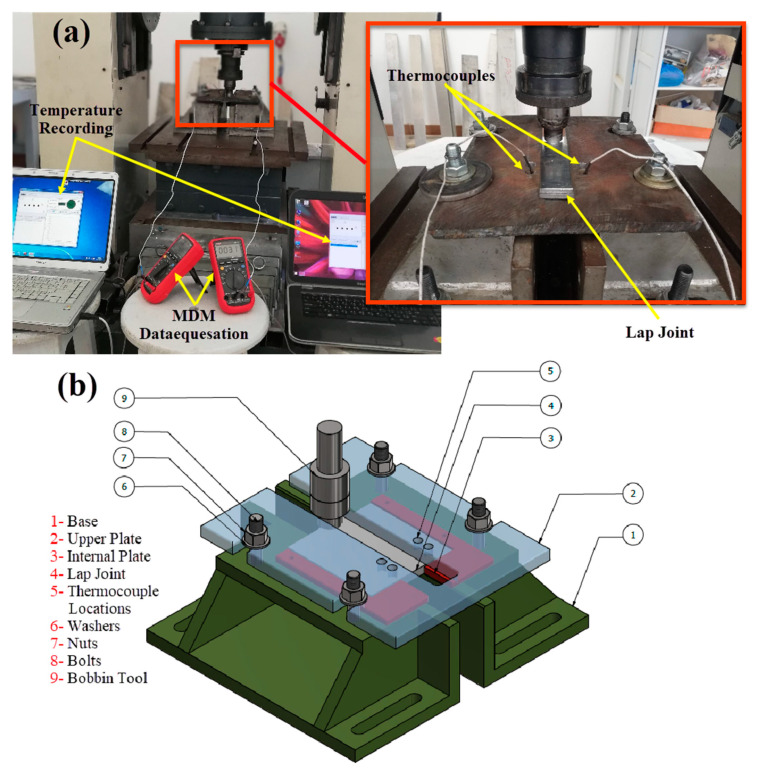
BT-FSW fixture setup configuration to weld AA1050-H14 in similar lap joints at the different welding parameters. (**a**) overview the BT-FSW using this fixture and (**b**) the 3D drawing with identification of all components.

The BT-FSW was carried out at various travel speeds “Ts” of 200, 400, 600, 800, and 1000 mm/min using the three different pin geometries (Cy, Sq, and Tr), and constant rotation speed “Rs” of 600 rpm at 0 tilt angle. Table 3 summarizes the BT-FSW process parameters. BT-FSW similar lap joints were carried out using the FSW machine (EG-FSW-M1) [21,22,23,24,25,26]. Adaptive fixture was designed and fabricated from steel for BT-FSW purposes. Figure 3a shows overview the BT-FSW using this fixture and Figure 3b shows the 3D drawing with identification of all components.

The generated temperatures in the stirring zone, advancing side, and retreating side during the BT-FSW process were measured using two techniques. The temperature distribution along the lap joint during BT-FSW has been measured at several points. The temperature at the weld surface behind the tool was measured using an infrared thermometer (Quicktemp 860-T3, Testo Company—Berlin, Germany). The temperature was measured at different stirring zones during the traveling of BT at the centerline during welding specimens for all processing parameters in terms of various traverse welding speeds and different pin geometries. The mean values were recorded for each welding condition for comparison purposes. A special setup was created using Modern Digital Multimeter (MDM) model (UT61B—Zhejiang, China) with thermocouple type “K” in order to measure the temperature at the AS and RS as given in Figure 3a. With MDM device, two thermocouples were used to collect the temperatures by placing them in two holes close to the weld pass in the heat-affected zone (HAZ) with 3 mm diameters and 3 mm depth drilled on both: retreating side and advanced side.

**Figure 4:**
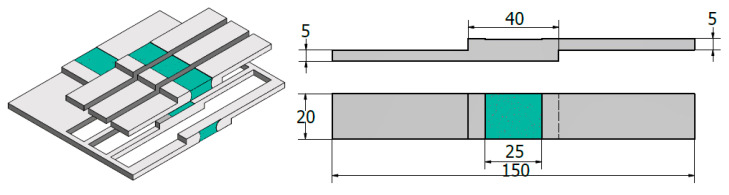
Schematic of the tensile-shear test sample (all dimensions in mm).

**Figure 5:**
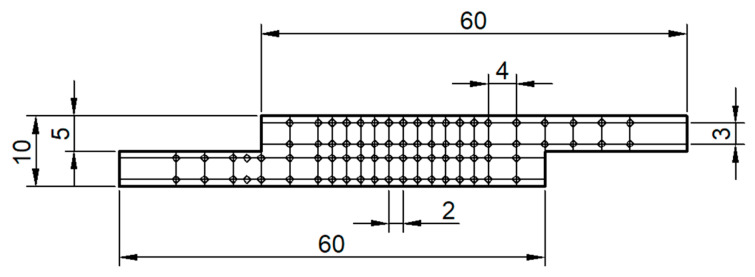
Schematic of measurement points of Vickers hardness profiles on the cross-section perpendicular the welding direction (all dimensions in mm).

The produced BT-FSW joints were evaluated at the beginning via visual inspection. Then, the transverse cross-section of welds was macroscopically examined after applying the standard metallographic grinding and polishing. The polished surface was then etched for about 180 s in a solution of 5 mL nitric acid, 3 mL hydrochloric acid, and 2 mL hydrofluoric acid in 190 mL distilled water. Furthermore, the tensile-shear test specimens were cut along the joint’s transverse directions with the dimensions as shown in Figure 4. The test was carried out using Instron tensile test machine (Instron-4208-300 kN capacity, Norwood, MA, USA). The velocity of the moving head of the machine was 0.05 mm/min at room temperature. Additionally, the fractures parts were investigated using SEM-Quanta FEG 250 (FEI company, Hillsboro, OR, USA). Finally, the hardness profiles through three lines on transverse cross-sections of the BT welded joints were measured to evaluate the hardness values on the upper, middle, and lower parts of the weld and surrounding regions (Figure 5) using Vickers hardness tester (HWDV-75, TTS Unlimited, Osaka, Japan) under 0.5 kg load for 15 sec dwell time.

## 3. Results and Discussions

### 3.1. Temperature Measured at Weld Center

**Figure 6:**
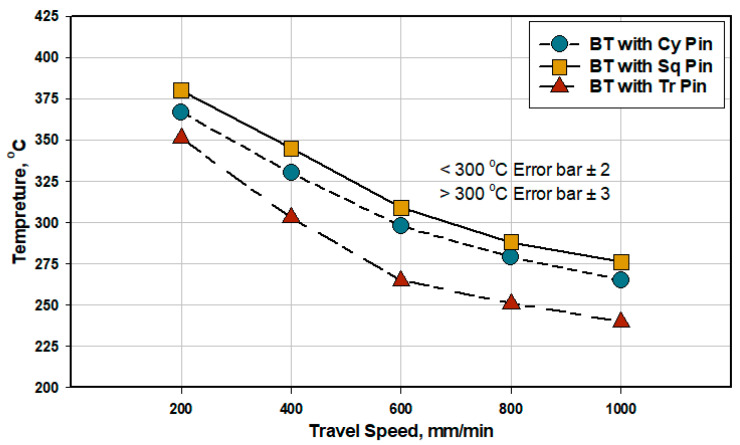
Effects of BT pin geometries and BT travel speed on the welding temperature in the stir zone to produce 10 mm AA1050-H14 similar lap joints.

The mean values of the measured temperature during the welding process were recorded and plotted versus different BT-FSW travel speeds of 200, 400, 600, 800, and 1000 mm/min using the three different pins Cy, Sq, and Tr at a constant rotation speed of 600 rpm and 0^o^ tilt angle as shown in Figure 6. Among all the produced 10 mm similar AA1050-H14 lap joints, the Sq pin geometry shows the highest stir zone temperature compared to the other two pins, Cy and Tr, whereas the Tr pin gives the lowest stir zone temperature at the various applied travel speeds from 200 to 1000 mm/min using BT-FSW, Figure 6. BT with square pin geometry produces higher weld temperature around 380 °C at 200 mm/min due to its surface shape that creates more friction heat during BT-FSW. The highest temperatures are 380 °C, 367 °C, and 351 °C, at the travel speed of 200 mm/min using BT with pin geometry of Sq, Cy, and Tr, respectively, whereas at the fast weld travel speed (1000 mm/min), the lowest temperatures are 276 °C, 265 °C, and 240 °C for the Sq, Cy, and Tr pins, respectively. Additionally, it can be seen that as the welding travel speed increases, the recoded sir zone temperature decreases for all the used pin geometries. This happens due to the reduced heat input per unit length and dissipation of heat over a wider region of the workpiece at a higher welding speed. In fact, the introduced temperature values in the stir zone during the FSW expresses the amount of heat input. The decrease of FSW heat input in the stir zoon as a function of increasing travel welding speeds was reported in many research with different weld joints for different materials [24]. The effects of pin geometry on the generated heat during the BT-FSW are clear (Figure 6). First, for the triangular pin, the frictional area between the pin surface and the workpiece material is limited to near the three edges, which is very smaller than that of the Cy and Sq pin geometries. Since a larger frictional area must generate a larger amount of friction heat, the friction heat generated by the triangular profile should be smaller than that by the Cy and Sq geometries. Second, for Tr and Sq pins, the weld temperature is directly proportional to the number of edges of the pin profiles because the increasing of edges leads to increasing of the frictional area in such a way that the weld temperature in the geometries increases from the Tr to Sq pin geometry during BT-FSW.

### 3.2. Recorded Thermal Cycles and Temperatures at the AS and RS 

**Figure 7:**
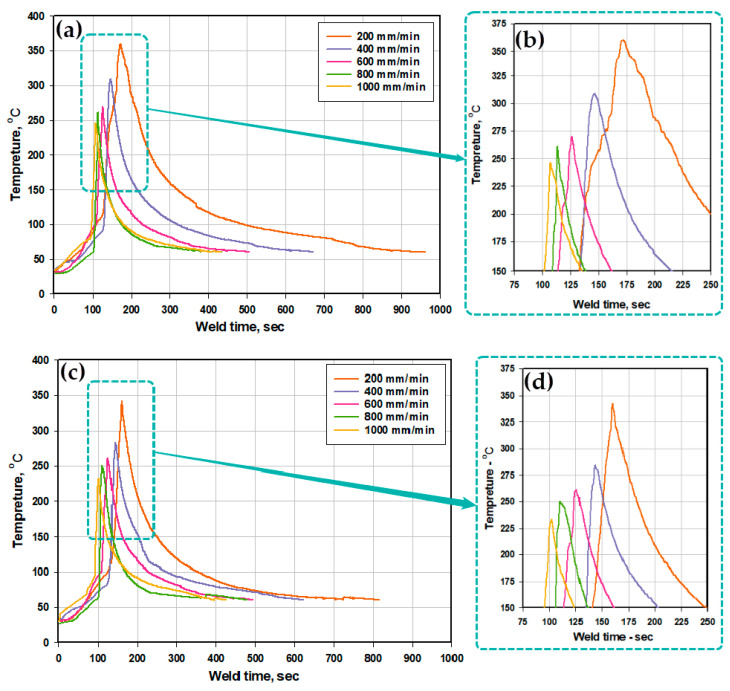
An example of the recorded temperature profiles obtained with the embedded thermocouples during BT-FSW of aluminum using the Sq pin using a constant rotation speed of 600 rpm at (**a**,**b**) advancing side and (**c**,**d**) retreating side.

The thermal cycles at both the AS and the RS in terms of the measured working temperatures as a function of time at the different welding speeds during the BT-FSW using the Sq pin are recorded with the embedded thermocouples and plotted in Figure 7a,c, respectively, with their associated enlarged areas for the overlapped peaks in Figure 7b,d, respectively. It should be mentioned here that the same trend of thermal cycle curves was obtained during the BT-FSW of AA1050-H14 using the Cy and Tr pins at the same applied welding conditions with a little difference in the recorded peak temperatures at each travel speed. In the current work, the BT-welding process can be divided into three steps. First, by inserting the tool to the center line of the lap joint and applying a rotation speed of 600 rpm and slow travel speed of 20 mm/min, the working material can be heated to a certain temperature that allows the initial softening of the material. Second, by applying a holding time of around 15 secs to heat the plates under the same rotation speed of 600 rpm, the temperature of the first and second steps is lower than 100 °C. Third, applying the actual travel speed to start achieving the welding path, the temperature of these steps is increasing gradually to the peak temperature. Finally, the process ended by air-cooling the workpiece with loss in the heat gradually. These steps appear in Figure 7. Using BT-FSW with Sq pin geometry, the maximum temperature experienced about 360 °C at the 200 mm/min welding speed at the AS that slightly higher at the RS. The minimum temperature experienced about 206 °C at the RS that slightly smaller at the AS using Tr pin geometry.

**Figure 8:**
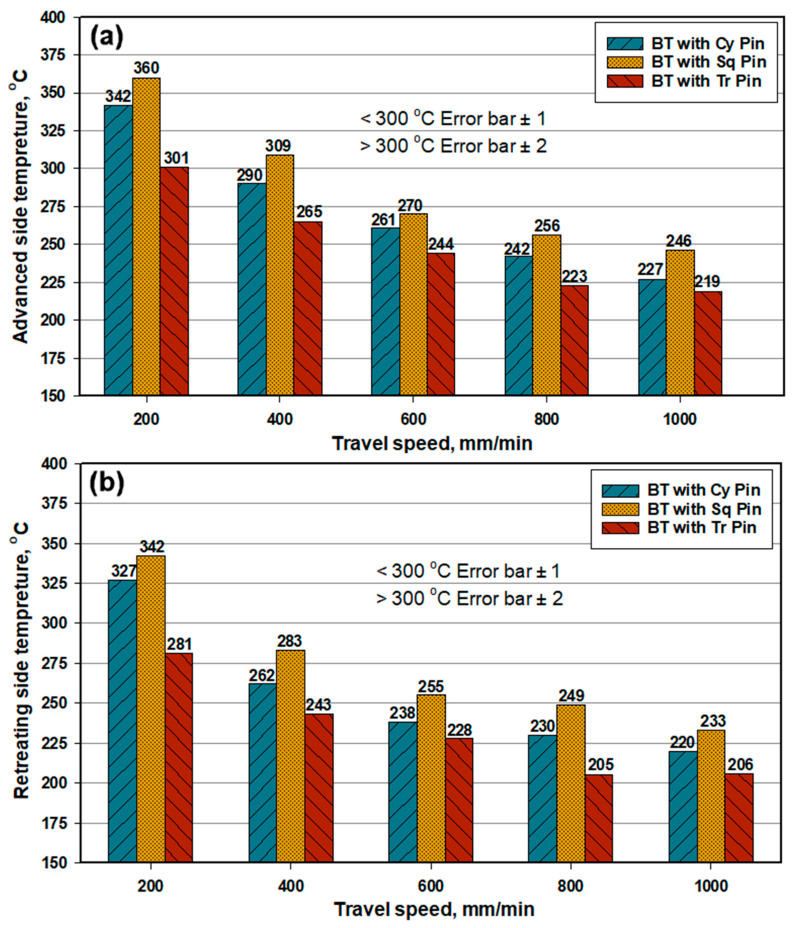
Maximum temperature measured at (**a**) the advancing side and (**b**) the retreating side with different pin profiles.

Figure 8 shows a comparative histogram for the temperatures measured during BT-FSW using the different pin geometries and at the different welding speeds for both the AS (Figure 8a) and RS (Figure 8b). The top surface temperature at both the AS and RS during BT-FSW decreases with increasing travel speed. This can be attributed to the reduction in the number of rotations per unite length by increasing the welding speed at a constant rotation rate. For example, 3 rev/mm at 200 mm/min is reduced to 0.6 rev/mm at 1000 mm/min. This significantly reduces the amount of heat generated by increasing the welding speed and also allows fast cooling after welding. On both sides, the BT with Sq pin generated a higher temperature, and the triangular pin generated the lowest temperature; this trend is the same trend for the temperature produced in the weld center at the top surface. The temperature increases along the direction of the weld and towards the weld center, and the weld temperatures recorded on the retreating side are slightly lower than that at the advancing side. This happens because the advancing side in the BT-FSW process is the location from which the softened solid material starts to flow around the tool pin plunged into the material, which moved from the front of the tool to the back of the tool to achieve the joint behind the tool in the solid state. The AS represents the start point for material stirring that requires the highest torque to achieve the required plastic deformation and heat to soften and move the material around the tool. Thus, it is expected to have a higher temperature at this side than the RS at which the material is left behind the tool and starts cooling.

### 3.3. BT-FSW Torque

**Figure 9:**
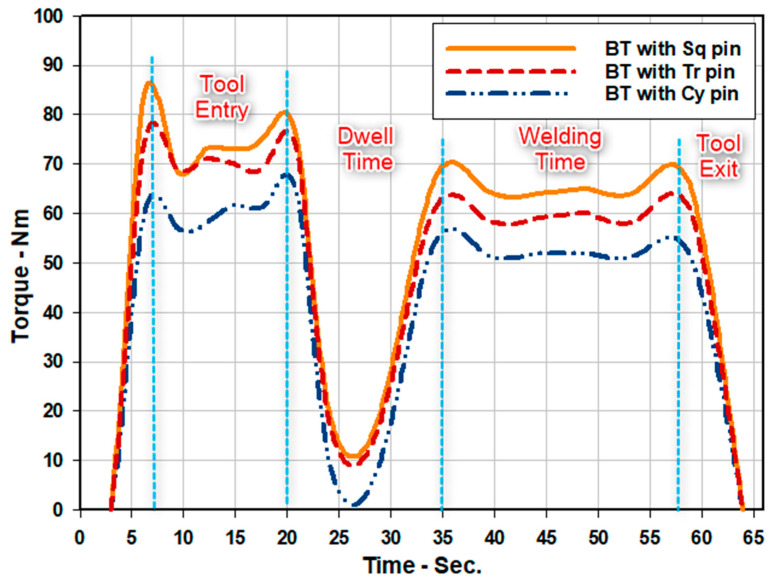
Torque during BT-FSW with different pins at 200 mm/min and 600 rpm.

There are many parameters controlling the stirring process in BT-FSW [2]. One of these is the torque. However, few works are developed to study and predict the torque during FSW, which can be used to optimize the tool design as well as the process [27]. Hence, predicting, monitoring, and controlling the BT torques are highly important to predict the tool life. In fact, the torque value given in the monitor of the full-automatic FSW machine in the present work can be used as an indicator for the resistance of materials to move around the pin during stirring. Figure 9 shows the measured torque values during BT-FSW at a travel speed of 200 mm/min and rotation speed of 600 rpm using the different pin geometries. The different regions of torque curves obtained during the BT-FSW process are tool entry, dwell time, welding achievement, and tool exit. At tool entry, the BT moves forward to touch the cold workpiece, and then the torque sharply increased to reach maximum values for all the used pins. In the dwell time region, the values of torque decrease to the minimum value of 10 Nm as a result of softening the stirring material around the tool. During the welding process, the torque increases sharply again as the material highly resists the rotating pin in the stir zone. The resistance of materials to move around the tool increases with increasing sharp pin edges. The highest value of measured torque (66 Nm) was obtained using BT with Sq pin geometry whilst, the lowest value (52 Nm) is given using the Cy pin. Finally, the torque decreases sharply in the tool exit region as a result of BT leaving the welded workpiece.

**Figure 10:**
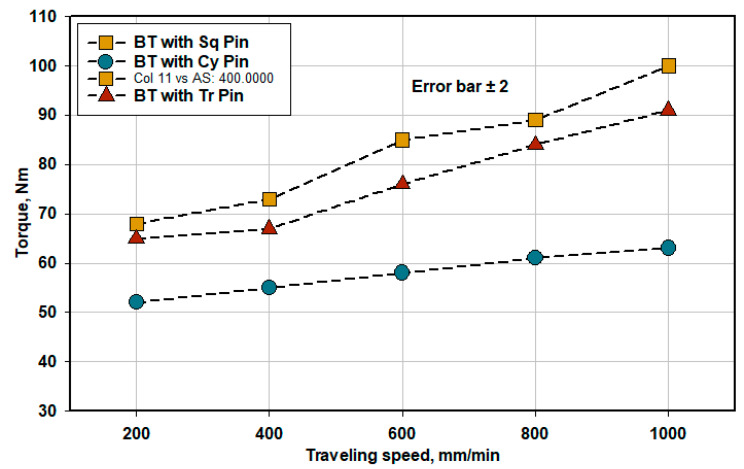
Torque during BT-FSW of AA1050-H14 with various pin geometries.

The torque value was recorded for the used three pin geometries as a function of travel speed and plotted in Figure 10. It can be seen that as the travel speed increases, the measured torque values increase for all the pin geometries. By increasing the welding travel speed, the heat input decreases. Thus, the torque value increases due to the difficulty of material flow [6]. Moreover, material flow with Cy pin is easier during the BT-FSW process compared to the other two used pins. On the other hand, the tool faces more resistance with the Sq, and Tr pins, especially at higher welding speeds due to the colder material.

### 3.4. Visual Inspection

**Figure 11:**
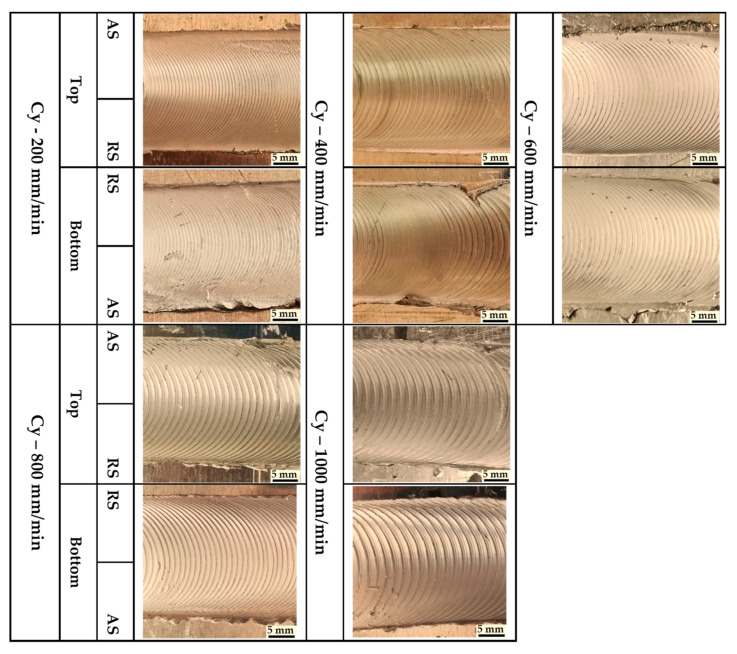
Top and bottom views of BT-FSW joints using Cy pin.

**Figure 12:**
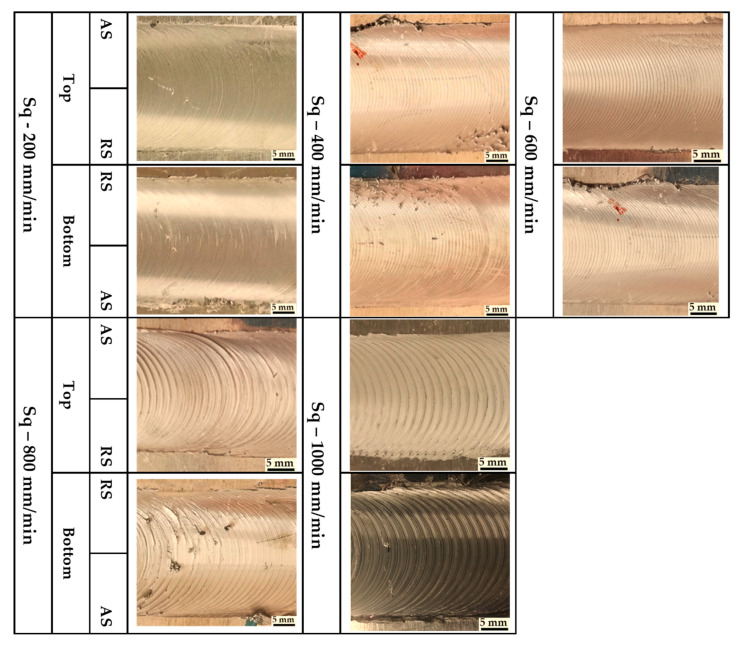
Top and bottom views of BT-FSW joints using Sq pin.

**Figure 13:**
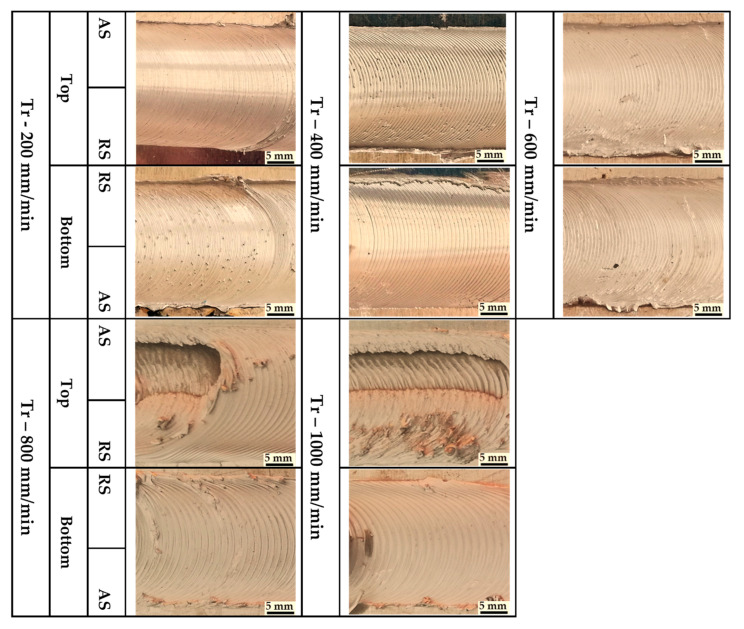
Top and bottom views of BT-FSW joints using Tr pin.

**Figure 14:**
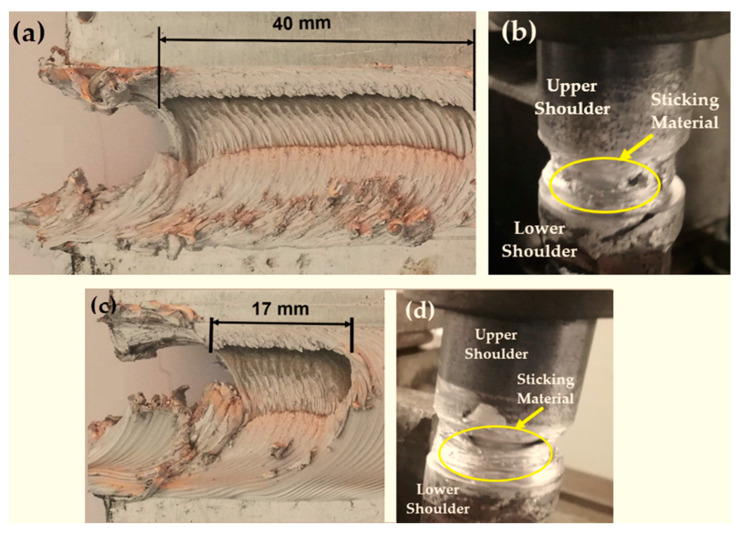
BT-FSW uncompleted joining defect and stick material around the BT using Tr pin at (**a**,**b**) 800 mm/min, (**c**,**d**) 1000 mm/min.

**Figure 15:**
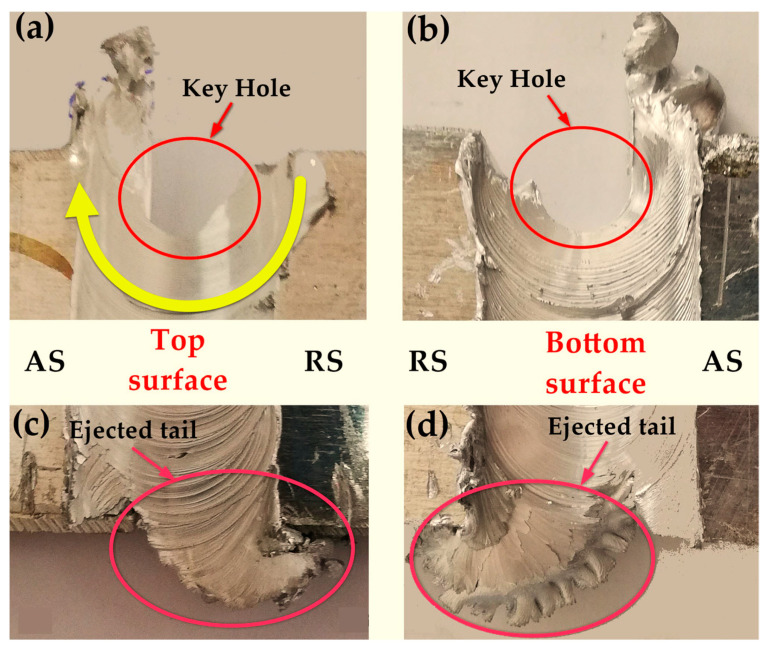
Top view of the BT-FSW of aluminum at start and end region; (**a**,**b**) the exit regions; (**c**,**d**) the entry regions.

Three groups of AA1050-H14 Al alloy similar lap joints were friction stir-welded using BT. Two welding parameters, pin geometry and travel speed, were considered with keeping the other parameters constant. The pin geometries were Cy, Tr, and Sq, and the travel welding speeds were varied from 200 to 1000 mm/min. Both the upper and the lower surfaces of the welded laps were visually inspected. No defects were observed for the first and the second welded lap joints groups using the Cy and Sq pins at all the applied welding speeds, as can be seen from the top and bottom surface views illustrated in Figure 11 and Figure 12, respectively. The third group of BT-FSW lap joints welded using Tr pin showed defect-free joints at 200, 400, and 600 mm/min travel speeds as given in Figure 13, whereas at 800 and 1000 travel speeds, uncompleted joints were observed on the joint top surfaces due to appearance of a tunnel defect. A tunnel may be formed during FSW due to insufficient heat input. The decrease in the stir zone temperature leads to a decrease in materials plasticity and hence hinders the flowability of the stirring material around the tool. Furthermore, the length of the uncompleted joining defect increases with increasing the travel speed from 800 to 1000 mm/min, as shown in Figure 14a,b. Some moving layers tend to stick on the rotating tool using Tr pin geometry, as given in Figure 15c,d.

Figure 15a,b shows the keyhole in the exit regions of the BT-FSW joints after the BT exits the joints. The BT leaves the joint by a disruption at the end of the welded pass. The arrows on the top (Figure 15a) and bottom (Figure 15b) surfaces show the last vestige of the shoulder on the top and bottom surfaces. The material is transported from the retreating side to the advancing side before starting the weld disruption. However, the stirring mechanism is not able to continue primary stirring from the advancing side to the retreating side, as the stirring material accumulated in the advanced side [28]. Figure 15c,d presents the shape of material flow of the entry regions for the bobbin welds on the upper and lower surface, respectively. The entry defect consists of an ejected tail and is produced on the retreating side. In the early step of the BT entering the workpiece, the material flows from the retreating side to the advancing side are disrupted, and the plasticized mass flows outwards of the joint. The main reason for the material ejection in the entry zone is the free surface at the trailing edge and the absence of the solidified mass to block the material flow outwards [29].

### 3.5. Macrostructure of BT-FSW Lap Joints

**Figure 16:**
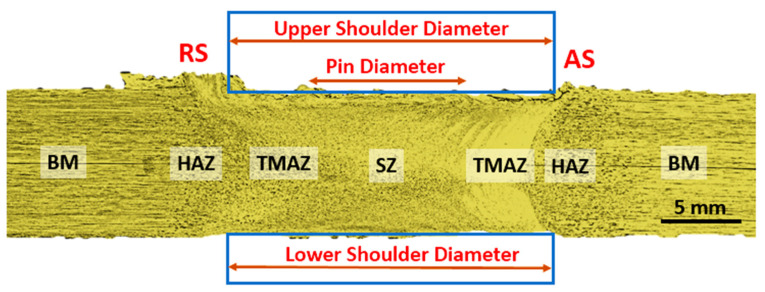
Macrograph analysis of BT-FSW of lap joints welded at 600 rpm and 200 mm/min.

**Figure 17:**
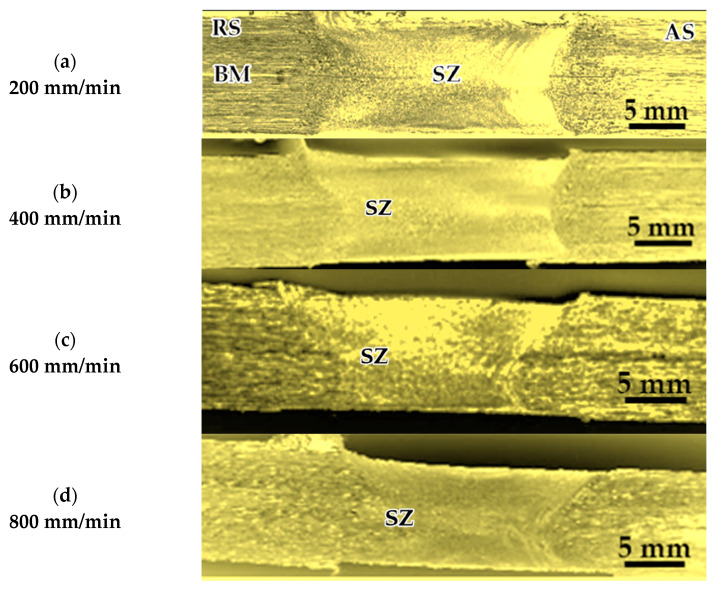

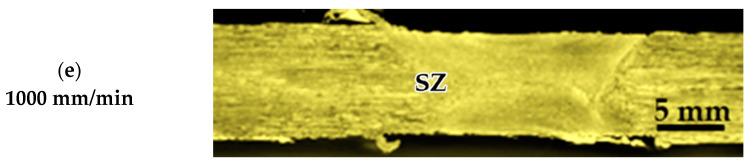
Macrographs of transverse cross-sections of lap joints produced at 600 rpm rotation speed and different travel speeds of (**a**) 200, (**b**) 400, (**c**) 600, (**d**) 800, and (**e**) 1000 using BT with Cy pin.

**Figure 18:**
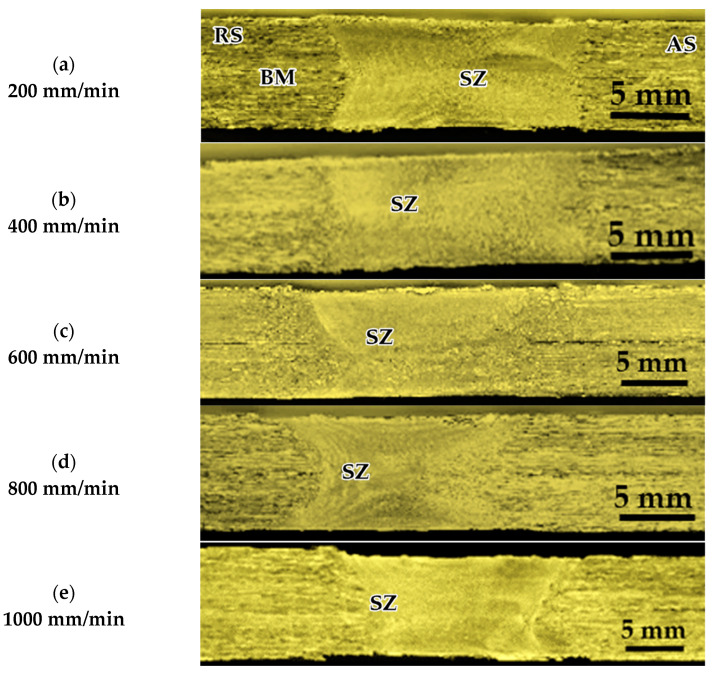
Macrographs of transverse cross-sections of lap joints produced at 600 rpm rotation speed and different travel speeds of (**a**) 200, (**b**) 400, (**c**) 600, (**d**) 800, and (**e**) 1000 using BT with Sq pin.

**Figure 19:**
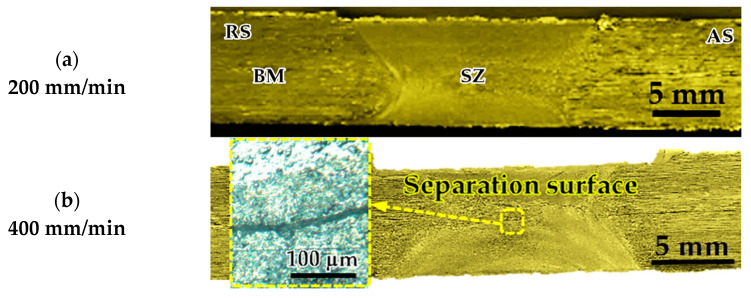

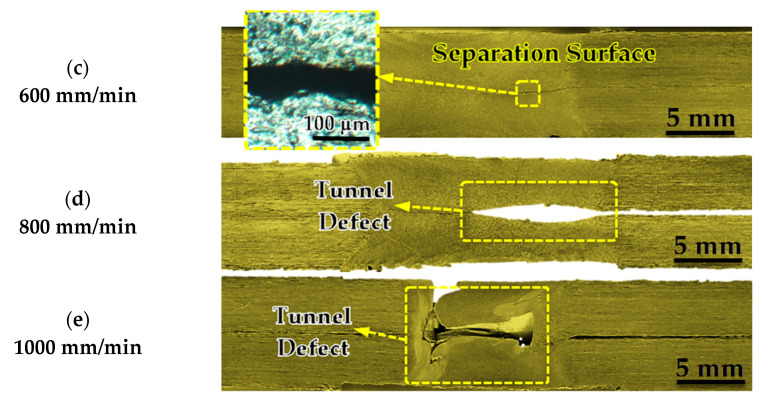
Macrographs of Transverse cross-sections of lap joints produced at 600 rpm rotation speed and different travel speeds of (**a**) 200, (**b**) 400, (**c**) 600, (**d**) 800, and (**e**) 1000 using BT with Tr pin; (**b**,**c**) separation surface defects; (**d**,**e**) tunnel defects.

Figure 16 shows the cross-sectional macrostructures of the BT-FSW lap joints welded at a rotation speed of 600 rpm and travel speed of 200 mm/min using Cy pin geometry. The different regions in welded joints, BM, HAZ, TMAZ, and SZ, can be easily featured. As seen in Figure 16, a sharp transition between the BM and the SZ occurs on the advancing side, while a more diffuse transient region is present on the retreating side of the welded joints. This has been attributed to the different flow behaviors and the degree of plastic strain/deformation of the material on both sides of the rotating and traveling tool [1]. The macrostructures of the transverse cross-sections of the AA1050-H14 lap joints welded using Cy, Sq, and Tr pins at various travel welding speeds (200, 400, 600, 800, and 1000 mm/min) and at a constant rotation speed (600 rpm) were investigated perpendicular to the welding directions and presented in Figure 17, Figure 18 and Figure 19, respectively. All cross-sections BT-FSWed at the applied travel speeds using Cy and Sq pin are defect-free, as shown in Figure 17a–e and Figure 18a–e, respectively. The relationship between defects observed in the welds and tool pin geometry is important to analyze. Many researchers [29,30] reported that due to the inappropriate movement and insufficient plasticization of material in the stir zone, the joints show various defects such as kissing bonds, voids, and tunnel defects. It was observed that the BT-FSWed joints obtained using triangular pin geometry showed the presence of tunnel defect, Figure 19. In lap joints produced with Tr pin at 400 and 600 mm/min, separation surface between the upper and lower plates at the joint middle transverse cross-section is shown in Figure 19a,b. Tunnel defects are more pronounced, especially in joints produced at 800 and 1000 mm/min travel speeds. The tunnel defect in the cross-sectional area at 1000 mm/min is larger than that obtained at 800 mm/min, as shown in Figure 19c–e. In general, the defect area increases with increasing travel speed. This is likely due to the decrease of weld temperature, which results in a lack of stirring action, and then promotes the chance of tunnel formation [31,32]. Similar results were also obtained by Hussain et al. [30] by using a triangular tool for FSW of AA6063 butt joints.

### 3.6. Mechanical Properties of BT-FSW Joints

**Figure 20:**
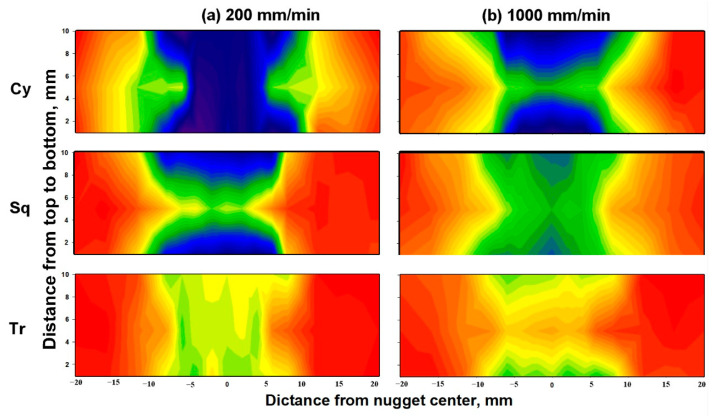
Hardness maps obtained at the transverse cross-section of the BT-FSWed lap joints at (**a**) 200 mm/min and (**b**) 1000 mm/min, using different pin geometries (Cy, Sq, and Tr).

Figure 20 shows the Vickers hardness maps obtained along the transverse cross-sections of the BT-FSW lap joints welded at a rotation speed of 600 rpm and at welding speeds of 200 and 1000 mm/min, using Cy, Sq, and Tr pin geometries. Generally, the hardness profile indicates the typical hardness profile for the strain hardened aluminum alloys that is characterized by a softened region of reduced hardness at the weld nugget; however, a significant effect can be observed in the hardness profile either due to the tool pin geometry or the welding speed. The hardness is mainly affected by the thermal cycle experienced and the maximum temperature. This can be observed in the width of the softened region and the level of hardness reduction in the weld zone. The highest reduction in hardness is occurred at 200 mm/min welding speed and using the Cy BT, as can be observed from the hardness maps that represented in blue color with a hardness of about 24 Hv. Using the same Cy pin at a higher welding speed of 1000 mm/min has reduced the level of hardness reduction, and the hardness reached about 27 Hv at the middle section. The lowest hardness reduction is achieved using the Tr BT at 1000 mm/min, and hardness reached about 29 Hv at the middle section, as can be noted from the orange color of the map. On the other hand, at the same level of hardness, the width of the softened region increases as the thermal cycle reaches higher temperatures for a long time, which occurred here at the low welding speed of 200 mm/min using the Sq tool pin. If, for example, one measures the width at a hardness value of 27 Hv, which is represented by the green color in the maps, it can be observed that the width of the softened region is the maximum at a welding speed of 200 mm/min using the Cy BT and the minimum at the welding speed of 1000 mm/min using the Tr BT. It can also be noted that at all the welding conditions, the hardness is lower at the shoulder surfaces, either top or lower shoulder, due to the maximum heat generated. The results obtained here are consistent with the reported in the literature during FSW of the strain-hardened aluminum alloys such as AA5083 and AA1050.

**Figure 21:**
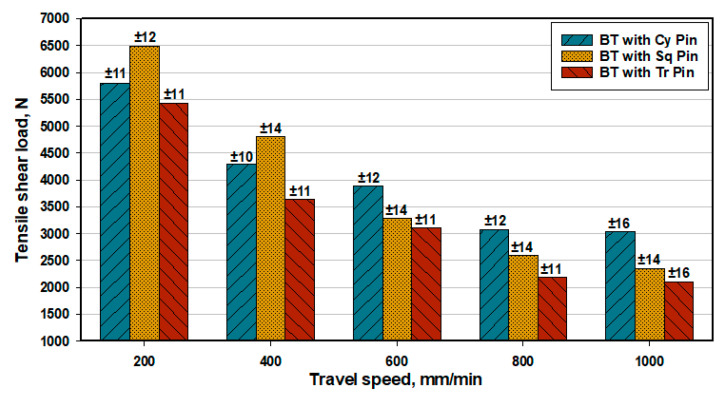
Tensile-shear loads of BT-FSW lap joints at various travel speeds using different pin geometries.

The tensile-shear load of BT-FSW lap joints using the different pins (Cy, Sq, and Tr) at various travel speeds of 200, 400, 600, 800, and 1000 mm/min, at a constant rotation speed of 600 rpm, was plotted in Figure 21. For all the applied pin geometries, the tensile-shear load increases with decreasing travel speed, reaching its peak value at 200 mm/min. The highest tensile-shear load values of the AA1050 similar lap joints were obtained at 200 mm/min welding travel speed. These values are 6491, 5806, and 5419 N for the applied pin geometries: Sq, Cy, and Tr, respectively. In CT-FSW, the pulsating action (pulse/s) (pulse/s = rotational welding speed in one second × number of flat faces) during the stirring process in FSW is a function of rotational speed and the number of flat faces of the pin geometry [29]. Goyal et al. [33] studied the effect of pin geometry on the pulsating action of the material flow around the pin in the stir zone for Al-Mg4.2 at welding conditions of 1200 rpm and 65 mm/min. They concluded that the cylindrical tool does not promote a pulsating action, whilst the triangular and the square pin geometries generate 60 and 80 pulse/s, respectively. Additionally, Elangovan et al. [17] reported that for FSW of AA2219 using different pin geometries, the Sq pin produced more pulse/s compared to the Tr pin. At the rotational welding speed of 1200 rpm, the pulses/sec values were 80 and 60 for the Sq and Tr pin geometries, respectively. The pin geometry of the BT-FSW is mainly responsible for softening and transporting the welded material during the joining process. The movement of plasticized material in the stir zone is controlled by two effects. The first effect is extrusion, where the pin pushes the plasticized material behind it. Additionally, the second effect is the rotary movement of the pin, which promotes the required thrust to ease the material flow [29]. According to the current welding conditions of 600 rpm rotation speed and different pin geometries, the Sq pin geometry produced 40 pulse/s and the Tr pin geometry produced 30 pulse/s, while no such pulsating action was observed in cylindrical pin geometry. The lap joints made with Cy and Tr showed lesser joint strength as compared to other welded lap joints with Sq pin (Figure 21), due to the lack of pulsating action and vertical flow of plasticized material in the stir zone. In the case of Tr pin geometry, improper consolidation of material near the pin base leads to the formation of separation surface 400 and 600 mm/min (Figure 19b,c). Additionally, there is a tunnel defect in the nugget zone at the welding condition of 800 and 1000 mm/min (Figure 19d,e), which consequently deteriorates the joint strength despite hardness increase. In addition, the lap joint welded at 200 mm/min using Tr and Cy pin geometries shows lower tensile-shear load compared with that produced using Sq pin geometry due to the of top and bottom surface roughness of the welded joints and the presence of weld protrusions.

**Figure 22:**
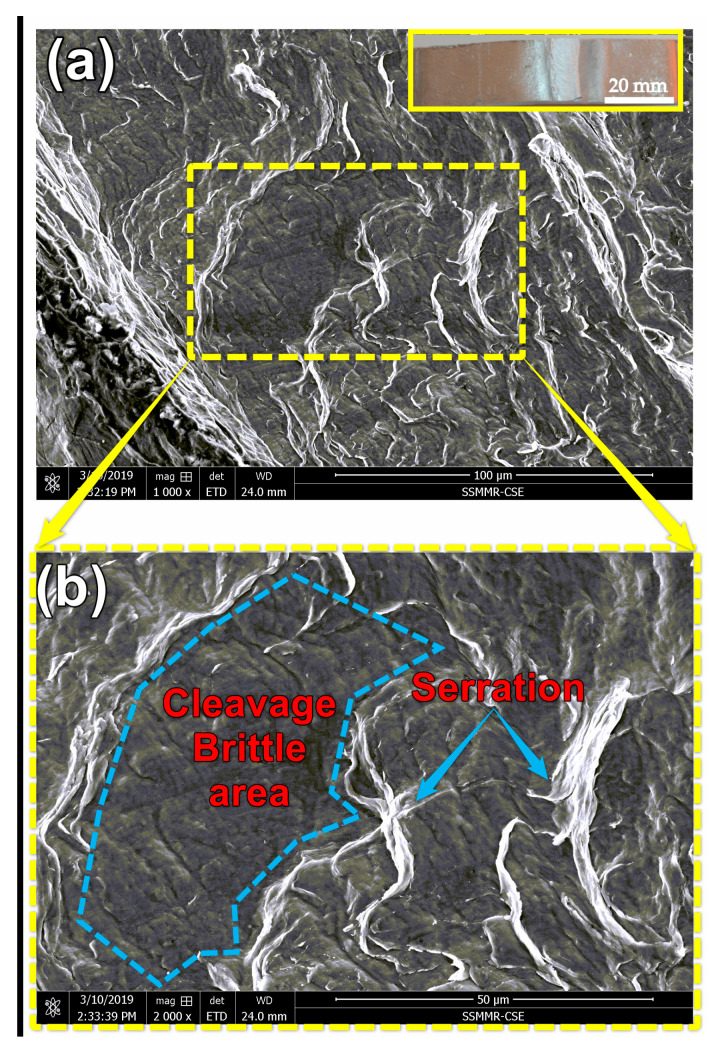
(**a**) The fracture morphology of the BT-FSW lap joint welded at travel speed of 200 mm/min and rotational speed of 600 rpm using Sq pin and (**b**) at a higher magnification.

**Figure 23:**
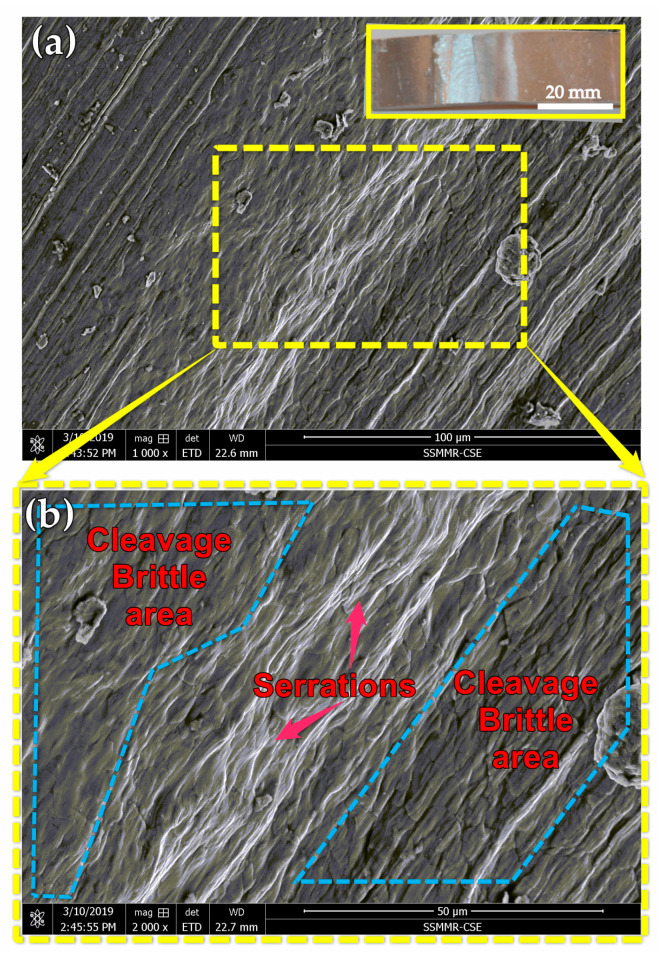
(**a**) The fracture morphology of the BT-FSW lap joint welded at a travel speed of 1000 mm/min and rotational speed of 600 rpm using Sq pin and (**b**) at a higher magnification.

Figure 22 and Figure 23 show typical fracture morphologies of the BT-FSW lap joint using an Sq pin at travel speeds of 200 and 1000 mm/min. It can be observed that the fracture surface of investigated fracture surfaces of the samples consists of mixed brittle and ductile modes. Figure 22 shows the fracture surface of BT-FSW at 200 mm/min, where ductile features as serrations-like areas are shown mixed with small cleavage fracture areas. These features indicate that the fracture mode is more ductile. On the other hand, the fracture mode of the joint produced using 1000 mm/min is mainly brittle, as shown in Figure 23. There is more cleavage faceted area, little serrations, and no dimples on the fracture surface. The above features are confirmed with the FSW heat input and tensile-shear results.

## 4. Conclusions

BT-FSW of AA1050-H14 similar thick lap joints was carried out using different pin geometries, Cy, Sq, and Tr, at a rotation speed of 600 rpm, and welding speeds from 200 to 1000 mm/min. The following conclusions could be drawn:

1. Pin geometry and travel speed showed significant effect as processing parameters in the BT-FSW process as they affect the temperature at the weld center. Furthermore, the square pin leads to a higher temperature, and the temperature in the advanced side is higher than that in the retreating side at all welding conditions.
2. Increasing the travel speed increases the torque, and the highest torque values are associated with the square pin geometry.
3. BT-FSW using Cy and Sq pins produced defect-free welds at all welding conditions, whereas at travel speeds of 400 and 600 mm/min, using a Tr pin resulted in uncompleted joining. Furthermore, tunnel defects appeared at welding speeds of 800 and 1000 mm/min.
4. At all the welding conditions, the hardness values of the stir zone are lower than those of the BM.
5. The welding conditions of 200 mm/min and 600 rpm using BT with Sq pin produces sound thick lap joints of AA1050-H14 Al alloy with a higher tensile shear load.
6. Mixed brittle-ductile fracture mode is observed on the tensile-fractured FSW samples.

## Figures and Tables

**Table 1:** Chemical composition of AA1050-H14 Al alloy.

Composition, in wt.%							
Fe	Si	Cr	Zn	Mg	Cu	Mn	Al
0.50	0.25	0.1	0.07	0.05	0.05	0.05	Balance

**Table 2:** Mechanical properties of AA1050-H14 Al alloy.

Mechanical Properties			
Tensile strength, MPa	Yield Strength, MPa	Elongation, %	Hardness, HV
110	103	10	28–30

**Table 3:** BT-FSW process parameters of AA1050-H14 lap joints.

BT-FSW Process Parameters	
BT pin profile	Cylindrical pin, square pin, triangle pin
Rotation speed, rpm	600
Travel speed, mm/min	200, 400, 600, 800, 1000

## Data Availability

The data presented in this study are available on request from the corresponding author. The data are not publicly available due to the extremely large size.
